# Implementation of an Artificially Empathetic Robot Swarm

**DOI:** 10.3390/s24010242

**Published:** 2023-12-31

**Authors:** Joanna Siwek, Patryk Żywica, Przemysław Siwek, Adrian Wójcik, Witold Woch, Konrad Pierzyński, Krzysztof Dyczkowski

**Affiliations:** 1 Department of Artificial Intelligence, Faculty of Mathematics and Computer Science, Adam Mickiewicz University, Uniwersytetu Poznańskiego 4, 61-614 Poznań, Poland; jsiwek@amu.edu.pl (J.S.); bikol@amu.edu.pl (P.Ż.); witwoc@st.amu.edu.pl (W.W.); konpie1@st.amu.edu.pl (K.P.); 2 Institute of Robotics and Machine Intelligence, Faculty of Automatic Control, Robotics and Electrical Engineering, Poznan University of Technology, Piotrowo 3A, 60-965 Poznań, Poland; przemyslaw.siwek@put.poznan.pl (P.S.); adrian.wojcik@put.poznan.pl (A.W.)

**Keywords:** artificial empathy, swarm, fuzzy sets, similarity measure, open simulation, physical experimentation

## Abstract

This paper presents a novel framework for integrating artificial empathy into robot swarms to improve communication and cooperation. The proposed model uses fuzzy state vectors to represent the knowledge and environment of individual agents, accommodating uncertainties in the real world. By utilizing similarity measures, the model compares states, enabling empathetic reasoning for synchronized swarm behavior. The paper presents a practical application example that demonstrates the efficacy of the model in a robot swarm working toward a common goal. The evaluation methodology involves the open-source physical-based experimentation platform (OPEP), which emphasizes empirical validation in real-world scenarios. The paper proposes a transitional environment that enables automated and repeatable execution of experiments on a swarm of robots using physical devices.

## 1. Introduction

The paper describes research motivated by the application of artificial empathy algorithms in a swarm of mobile robots. The goal is to transfer the biological mechanisms of the human brain, such as empathy, to computer systems. This improves the quality of the robots by extending them with cognitive aspects, such as learning and adaptation mechanisms.

We present preliminary results of computer simulations that identified problems causing divergence between simulations and reality, such as significant resource consumption, which limits the number and range of parameters. This limitation prevented the effective evaluation of the algorithms. Therefore, we propose a solution to address the issue of discrepancies between the simulation environment and real experiments. Our solution allows for the verification of complex algorithms and offers universality, accessibility, standardization, and repeatability of results.

The construction of a platform that achieves those objectives requires intensive research. This paper presents the assumptions made and solutions used for the construction of two versions of mobile robot prototypes and an experimentation arena. Particular attention is paid to the possibility of modeling empathetic behavior in the swarm, which had a significant impact on many technical requirements for the devices built.

### 1.1. Motivation

In the field of robotics, the standardization and reproducibility of research results are primary challenges, particularly in the field of AI. Research outcomes often depend on various factors, such as hardware configuration, software version, or environmental factors. Simulators, such as CoppeliaSim [1], available in the field, may not accurately reflect reality. One issue in AI research is the significant centralization of the research and development process. Major academic institutions possess substantial resources, which limits opportunities for smaller universities and individual researchers to engage in state-of-the-art research.

One challenge in AI and swarm robotics research is the high entry barrier. Smaller research units may find it difficult to acquire the specialized tools and infrastructure required for this type of research. To address this issue, a proposed solution is to provide an affordable and standardized experimental environment for swarm robotics. Remote access to an environment enables experiments to be conducted without physical access to the test platform, enhancing the potential for international and interdisciplinary collaboration. Cloud access in a pay-as-you-go model could reduce initial expenses on research infrastructure [2].

The transition from the research stage to industrial implementation highlights the challenge of manufacturing and testing swarms in operational conditions. Furthermore, there is a shortage of variable and modifiable test environments for swarms, particularly those with remote access capabilities. The restricted applicability of existing cloud-based testing platforms is also a concern, as demonstrated by the case of DeepRacer [2], which is exclusively designed for robot racing. Imperfections in existing robotics simulators, such as CoppeliaSim, may result in inaccurate representations of reality. Enabling the modification of the real test environment would greatly facilitate the testing of swarm solutions at an early stage of development, leading to the development of more effective products. For example, the DeepRacer platform has a race track that cannot be modified, which limits the number of testing scenarios. Remote access to an experimental platform with mobile robots would enable the testing of swarm algorithms without requiring physical access to the equipment. This approach could reduce capital costs for companies utilizing AI in swarm robotics solutions and redirect capital toward operational activities.

To address this problem, it is necessary to test algorithms in a standardized and predictable environment. This can be achieved by providing greater versatility and computational power than other commonly used tools, such as Kilobots [3]. To explore various swarm robot operation scenarios in different environments and ultimately improve efficiency, it is recommended to introduce a modifiable experimental platform. The creation of a highly automated experimental environment using physical devices could resolve issues that are inherent to simulation software. This would open up new research opportunities for scientists working on challenges related to robotics and artificial intelligence.

In summary, the use of advanced technologies and sensors in artificial intelligence research is essential. These innovations not only simplify and enhance the research process but also broaden the accessibility of AI technologies. Standardized experimental environments, particularly in the realm of swarm robot experiments, address issues related to result reproducibility, fostering international and interdisciplinary collaboration in AI.

The flexibility and universality of the experimental platform are crucial for algorithm testing in diverse environments, leading to the identification of more efficient solutions. The implementation of a highly automated experimental environment, grounded in physical devices, mitigates challenges associated with simulator imperfections and limitations of simulation software.

In addition, remote access to the experimental platform is a game-changer for researchers, as it allows them to conduct experiments without the need for physical presence. This not only streamlines research efforts but also facilitates educational activities in schools, overcoming barriers such as high costs and challenges associated with engaging in AI research. The integration of advanced technologies and the establishment of robust experimental frameworks are essential for advancing AI research and promoting its broader application.

This article presents the process of developing the concept of such an environment, introduces the proposed model of artificial empathy, and discusses the methodology and results obtained in simulation-based experiments and work on building two prototype versions of the solution.

### 1.2. Empathy Modeling

The human decision process relies highly on a person’s knowledge, intelligence, and experience. Emotional intelligence that constitutes a large part of general intelligence [4] plays a significant role in action-taking, especially involving cooperation [5]. In such a case communication is crucial, and a significant part of every message consists of signaling emotions and the inner state of the sender [5]. Empathy is the ability to put oneself in the “mental shoes” of another person, to understand their emotions and feelings [6]. It allows one to predict what kind of behavior can be expected from the target of empathy and to plan own actions accordingly. Since human reasoning is highly imprecise, one does not have complete information about the inner state of the empathy target and can only reason based on imperfect, highly subjective knowledge. When creating artificial intelligence systems, especially cooperative ones like swarms, omitting empathy seems to be wasteful. The attempt to transfer the concept of human empathy to artificial systems is called artificial empathy [7,8].

Human reasoning, particularly the aspect connected with emotions, is imprecise. Therefore, recreating decision mechanisms that include empathy requires tools that allow the model to understand imprecision. Empathy necessitates comparing the emotions and states of others to one’s own knowledge, thus requiring imprecise representations of those states. As complete information about the inner state of the empathy target is unavailable, deductions can only be made based on a signal that is not ideal and subjectively produced and understood. Fuzzy sets and linguistic variables offer useful solutions.

Artificial empathy is an increasingly popular research topic [9]. Its inclusion in various fields, including marketing ([10]) or robotics ([11,12]), brings many benefits to end users. The most common application of artificial empathy can be found in medicine [13,14,15]. Another application of artificial empathy is in computer games. The ability of AI systems to emulate empathy can help create more realistic characters and environments that allow players to feel part of the game world. Artificial empathy systems are also used in games to increase players’ empathy toward certain social groups or characters in the game. This allows players to better understand and identify with the characters, which in turn can influence their in-game decisions [16].

In the paper, we will deal with fuzzy agents, i.e., agents that decide with fuzzy knowledge [17]. The decision is whether an egoistic or empathetic action should be taken to achieve a shared goal. Egoistic action is focused on a local goal, compatible with the general goal of the swarm. Empathetic action is cooperative. The problem at hand is whether the swarm performance can be further optimized by integrating socio-psychological aspects of empathy in swarm control.

Agent actions are based on generalized knowledge, represented as a table of a fixed number of fuzzy sets, modeling action–consequence pairs. Fuzzy feature representation converts the collected information into membership degrees [18]. Resulting degrees are taken as a description of the agent’s state. Before an agent performs an action, its current state is assigned a reward, based on the similarity to known states and their outcomes. After the performed action, the state is assigned a realized reward, and is then remembered as “knowledge”. By replacing each value in the input data with their corresponding degree of membership to parameter realization we obtain a fuzzified set of labeled states [19].

Since empathy requires comparing the states of the agents, it is important to introduce similarity measures for fuzzy sets. From [20,21], we can ascertain that fuzzy sets are the proper tool to describe the similarity in such a situation. An agent will compare known, “experienced” states to a new one, for which the reward is not yet known or stored in any knowledge base available for the agent. The empathetic approach has great potential for use in swarms, as there are numerous opportunities for one agent to “help” another in achieving a shared goal.

### 1.3. Structure of the Paper

Simulation of swarm behavior is a crucial step before implementation on a target platform. However, simulations are often inaccurate and unrealistic, particularly for the empathetic swarm, due to the critical role of communication. In software simulations, it is not possible to reliably reflect problems or errors in signal transmission or hardware failures. This paper proposes a new approach to simulating empathetic behaviors in swarms using an open-source physical-based experimentation platform (OPEP). OPEP serves as an intermediate solution between software-based simulation and the target environment. The paper presents the architecture and current implementation of the platform.

The main purpose of this paper is twofold. The first one is to introduce a novel model of swarm control with the use of artificial empathy. The second is to establish an open-source physical-based experimentation platform, a cost-effective and reliable way to evaluate various models and scenarios in robotics, focused on modeling emphatic behaviors.

The paper is organized as follows: Section 2 presents materials and methods that include definitions of artificial empathy and its theoretical models. It also briefly reviews the available methods for conducting experiments in a swarm of robots. Section 3 contains the main results and introduces the idea of an empathetic swarm, presents simulation results of the proposed swarm model, and proposes the open-source physical-based experimentation platform. Section 4 concludes the obtained results and gives some ideas for further research.

## 2. Materials and Methods

### 2.1. Empathy Theory

Artificial empathy is the ability of computational systems to understand and respond to the thoughts, feelings, and emotions of humans [8]. Most definitions of artificial empathy describe it as the artificial version of human empathy [22]. Human empathy is said to consist of three components: emotional, cognitive, and motivational. Emotional and motivational empathy is more biological and allows for “automatic” responses to emotions elicited by internal or external factors. Cognitive empathy is more inductive—it allows an agent to understand the inner state of another agent, based on the signals they broadcast (e.g., expressions), and the situation they are in.

It is easy to notice that in swarms emotional empathy has no significant applications (for now, since robots do not yet commonly present or feel emotions [23]). Yet, there is a vast scope of possible applications of cognitive empathy, since cognitive empathy is connected with learning and deducing about behaviors in a certain environment. Knowledge sharing by cognitive empathy can help a swarm of robots learn effective behaviors faster.

In the literature, we can find three main models of artificial empathy, each of them created for different use cases.

1. Emotional and cognitive empathy model: The model was developed from medical and neuroscientific research of the human brain and has its justification in the brain structure. It assumes that empathy can be divided into parts: (1). responsible for recognizing and reacting to emotions; (2). a part responsible for cognitive, more logical, and deductive mechanisms of understanding the inner states of others [24].
2. Russian doll model: The model assumes that empathy is learned during human life—it resembles a Russian doll, with layers of different levels of understanding others. The first, most inner layers are mimicry and automatic emotional reactions, the next layers are understanding others’ feelings and the outer layers are taking the perspective of others, sympathizing, and experiencing schadenfreude [25].
3. Multi-dimensional model: This model assumes that we have four dimensions of empathy—antecedents, processes, interpersonal outcomes, and intrapersonal outcomes. Antecedents encompass the agent’s characteristics: biological capacities, learning history, and situation. Processes produce empathetic behaviors: non-cooperative mechanisms, simple cognitive mechanisms, and advanced cognitive mechanisms. Intrapersonal outcomes are to resonate or not with the empathy target, and interpersonal outcomes are relationship related [26].

Artificial empathy has found its applications in a growing number of fields and practical problems:

- Medical, e.g., in the detection of autism or Parkinson’s disease, depression treatment [13,15,27]
- Psychology, e.g., ethics, artificial companions [14,28];
- Marketing, e.g., personalized adverts [29];
- Entertainment e.g., games with high immersion or biofeedback [16,30,31];
- Education, e.g., empathetic tutors [32,33];
- Car industry [34].

Swarm applications also seem to open up an opportunity to effectively utilize empathy.

The idea of ‘swarm’ comes from nature—insects, animals, and people work together in cooperative groups to achieve goals that would be impossible to reach for a single agent. Examples are ants, bees, fish, etc.

The main characteristics of a swarm are a large number of agents, the absence of external control, and simple behaviors exhibited by individual agents. More advanced and complex swarm behaviors emerge automatically, based on environmental conditions and the capabilities of the agents.

One of the most important research areas of swarms is collective decision-making [35]. The problem has also an application in empathetic swarms, where the decision of which agent to help can be crucial in achieving the goal. An example can be found in [36].

Current research of empathetic swarm behaviors emerging automatically from broadcast and received signals include [37,38]. Also, the theory of mind is used to model collective behaviors of artificially modeled rescue teams [39,40]. The study by Huang et al. [41] considers a simulated swarm of a few caribou agents to escape from a superior wolf agent. The introduction of empathy in the form of an additional parameter (the distance from the chased caribou to the wolf), allowed for a significant increase in the number of learned successful escape strategies. This kind of approach is very limited—the decision process is automatic and based only on one parameter. It resembles more of an emotional contagion (one of the primitive levels of empathy), rather than a cognitive empathetic process. In our model, we would like to emphasize the role of learning, deduction, and experience in empathizing with other agents.

### 2.2. Available Experimental Environments

An analysis of various approaches and tools for conducting robot swarm experiments was conducted. The three main categories of available experimental environments are described below.

1. Stand-alone robots, allowing the construction and modeling of swarm behavior.
  - Kilobot [3]: This is a swarm-adapted robot with a diameter of 3.3 cm, developed in 2010 at Harvard University. It operates in a swarm of up to a thousand copies, carrying out user-programmed commands. The total cost of Kilobot parts was less than USD 15. Kilobots move in a vibration-based manner. In addition, they are capable of recognizing light intensity, communicating, and measuring the distance to nearby units. Currently, the project is not under active development, but it is still popular among researchers.
  - e-puck2 [42]: This is a 7 cm diameter mini mobile robot developed in 2018 at the Swiss Federal Institute of Technology in Lausanne. It supports Wi-Fi and USB connectivity. It has numerous sensors, including IR proximity, sound, IMU, distance sensor, and a camera. The project is being developed using open-source and open-hardware principles.
  - MONA [43]: This is an open-hardware/open-source swarm research robotic platform developed in 2017 at the University of Manchester. MONA is a small, round robot with a diameter of 8 cm, equipped with 5 IR transmitters, based on Arduino architecture.
  - Colias [44]: This is an inexpensive 4 cm diameter micro-robot for swarm simulation, developed in 2012 at the University of Lincoln. Long-range infrared modules with adjustable output power allow the robot to communicate with its immediate neighbors at a range of 0.5 cm to 2 m. The robot has two boards—an upper board responsible for high-level functions (such as communication), and a lower board for low-level functions such as power management and motion control.
  - SwarmUS [45]: This is a project that helps create swarms of mobile robots using existing devices. It is a generic software platform that allows researchers and robotics enthusiasts to easily deploy code in their robots. SwarmUS provides the basic infrastructure needed for robots to form a swarm: a decentralized communication stack and a localization module that helps robots locate each other without the need for a common reference. The project is not in development as of 2021.
2. Robot simulation software
  - AWS Robomaker: This is a cloud-based simulation service released in 2018 by Amazon, allowing robotics developers to run, scale, and automate simulations without the need to manage any infrastructure. It enables the creation of user-defined, random 3D environments. Using the simulation service, one can speed up application testing and create hundreds of new worlds based on templates that one defines.
  - CoppeliaSim [1]: This is a robotics simulator with an integrated development environment; it is based on the concept of distributed control. Each object/model can be individually controlled using a built-in script, plug-in, ROS node, remote API client, or another custom solution. This makes it versatile and ideal for multi-robot modeling applications. It is used for rapid algorithm development, simulation automation of complex processes, rapid prototyping and verification, and robotics-related education.
  - EyeSim [46]: This is a virtual reality mobile robot simulator based on the Unity engine, which is able to simulate all the main functions of RoBIOS-7. Users can build custom 3D simulation environments, place any number of robots, and add custom objects to the simulation. Thanks to Unity’s physics engine, robot motion simulations are highly realistic. Users can also add bugs to the simulation, using built-in simulated bug functions.
3. Comprehensive services including simulator and hardware platform.
  - AWS DeepRacer: This is a 1/18 scale fully autonomous racing car designed in 2017 by Amazon and controlled by Reinforcement Learning algorithms. It offers a graphical user interface that can be used to train the model and evaluate its performance in a simulator. AWS DeepRacer, on the other hand, is a Wi-Fi-enabled physical vehicle that can drive autonomously on a physical track using a model created in simulations.
  - Kilogrid [47]: This is an open-source Kilobot robot virtualization and tracking environment. It was designed in 2016 at the Free University of Brussels to extend Kilobot’s sensorimotor capabilities, simplify the task of collecting data during experiments, and provide researchers with a tool to precisely control the experiment’s configuration and parameters. Kilogrid leverages the robot’s infrared communication capabilities to provide a reconfigurable environment. In addition, Kilogrid enables researchers to automatically collect data during an experiment, simplifying the design of collective behavior and its analysis.

### 2.3. Fuzzy Sets and Their Similarity

In the presented paper, the agent’s states will be represented in the form of fuzzy sets. Fuzzy sets allow us to represent precise data, and since agents will communicate using non-ideal communication and will interpret the signals subjectively, fuzzy sets seem to be a proper tool to use.

Let $U=\{u_{1},u_{2},\ldots ,u_{n}\}$ be a crisp universal set. A mapping A:U→[0,1] is called a fuzzy set (FS) in *U* (denoted also by capital letters A, B, …). For each 1≤i≤n, the value $A(u_{i})$ ($A_{i}$ for short) represents the membership grade of $u_{i}$ in *A*. We say that fuzzy set *A* is a subset of fuzzy set *B* (A⊂B) if $A\left(u_{i}\right)\leq B\left(u_{i}\right)$ for all $u_{i}\in U$. Let F(U) be the family of all fuzzy sets in *U*.

A similarity measure of fuzzy sets is defined as a function s:F(U)×F(U)→[0,1] such that

(T1) for each (A,B)∈F(U)×F(U), we have s(A,B)=s(B,A),
(T2) for each (A,D)∈F(U)×F(U) and (B,C)∈F(U)×F(U) such that A⊂B⊂C⊂D we have
  (1) s(A,D)≤s(B,C),
(T3) for each X⊂U such that $\left(1_{X},1_{X^{c}}\right)\in F\left(U\right)\times F\left(U\right)$ we have $s(1_{X},1_{X^{c}})=0$ and $s(1_{X},1_{X})=1$.

This definition coincides with the classical one proposed by Xuecheng [48] (see also [49,50,51]). The higher measure values indicate a higher similarity of its arguments. It is usually assumed that all fuzzy sets are comparable by a given similarity measure. However, some similarity measures cannot be formally defined over the whole Cartesian product F(U)×F(U). The most commonly used similarity measure is the Jaccard index, defined as

(2) $$s\left(A,B\right)=\frac{A\cap B}{A\cup B}\;.$$

## 3. Results

### 3.1. Artificial Empathy of a Swarm

The general idea of artificial empathy in swarm applications comes from observing cooperative behavior in a group of agents (humans, animals) and realizing what types of knowledge and experience are needed to create successful behaviors and strategies.

In human cooperation, it is easy to see that taking the perspective of another person and trying to understand their point of view greatly improves cooperation and leads to better results. The two actions mentioned actually define cognitive empathy, i.e., drawing one’s own conclusions from the state broadcasted by another agent and the environment, thus anticipating the target’s behaviors based on inferred knowledge. The proposed model of an artificially empathetic swarm is based on how humans cooperate. People collect experience and knowledge and decide what action to take based on it. While cooperating, they take into account the experience and knowledge of others. Yet, it is impossible to access other people’s minds directly—one has to interpret signals sent from cooperating partners. These signals constitute largely of emotions [5]. After receiving a signal, the empathizer imagines what knowledge the signal represents, and what could be the possible consequences [6]. Based on the person’s own knowledge, they envision the state of the sender. Finally, an action choice is made—whether to use one’s own knowledge and capabilities or to combine efforts with others to improve performance.

**Figure 1:**
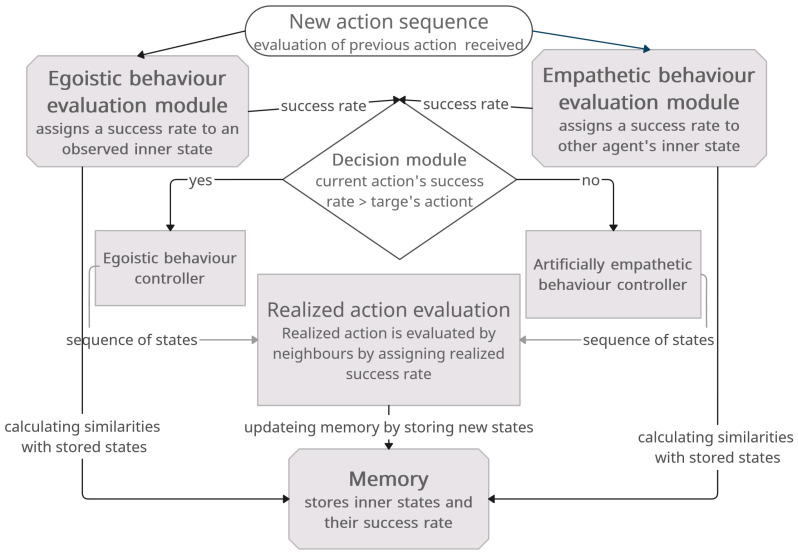
Swarm behavior control system with artificial empathy. Schema presents component modules for egoistic and empathetic control and behavior evaluations, decisions, and memories.

The proposed model consists of six parts: the module for evaluating egoistic behaviors, the module for evaluating artificially empathetic behaviors, the memory module for storing knowledge, the decision module for choosing the action type, and egoistic and artificially empathetic behavior controllers for choosing and executing particular actions. Modules are described in the following subsections and interactions between them are depicted in Figure 1.

#### 3.1.1. Egoistic Behavior Evaluation Module

The egoistic behavior evaluation module assigns a reward to an egoistic action, based on the state vector representing the state of the agent and perceived environment, and the knowledge of the agent. This state is defined as a fuzzy set:

(3) $$A=(a_{1},a_{2},\ldots ,a_{n})\;,$$

where $a_{i}\in \left[0,1\right]$, i=1,…,n are membership values, representing the satisfaction level of a *i*th state variable. All $a_{i}$ should not be correlated. The state should be updated in time. The state of the agent, which includes the perceived environment and the knowledge of the agent, is composed of two parts: emotional state (subjective knowledge) and cognitive state (objective facts). The first group may include information about the agent’s internal state (battery level, current action, call for help) and the second one may include information about the perceived environment (proximity to the goal, neighbors, obstacles).

Each of the states is evaluated to decide whether it can bring the agent closer to the goal. The state $A^{i}$ is assigned a reward $r(A^{i})$ based on the similarity to the known states and their outcomes, stored in memory and representing the agent’s knowledge. The agent has an initial set of states and their rewards, to be able to generalize in an unknown environment. Those first states can be understood as instincts or basic knowledge. The reward from the evaluated state $A^{i}$ is then calculated as

(4) $$r\left(A^{i}\right)=\frac{\sum_{j=1}^{m}s\left(A^{j},A^{i}\right)\cdot r\left(A^{j}\right)}{m}\;,$$

where *m* is the number of states and the rewards $\left(A^{j},r_{j}\right)$ stored in memory and s is a fuzzy similarity measure.

#### 3.1.2. Artificially Empathetic Behavior Evaluation Module

The target of empathy broadcasts its state, which the empathizing agent interprets based on their own knowledge. In our model, the empathizing agent (A) evaluates the target’s (B) state by comparing its broadcast state to the agent’s own knowledge. Reward r(B) is calculated using the same knowledge as the agent’s rewards according to (4). The rewards calculated for both the empathizing agent and the empathy target are used as decision parameters in choosing the agent’s next action.

#### 3.1.3. Memory Module

The memory module is responsible for storing the agent’s knowledge, obtained by performing actions. It is composed of state–reward pairs ($A^{i},r_{i})$. In the beginning, the agent only has initial states. With time and performed actions, new knowledge is added to this base. Namely, each action taken by the agent is evaluated and assigned a realized reward. The state representing the action and the corresponding reward are then added to the database and can be compared with other, new states to calculate similarity and assign rewards. Since the platforms used to implement the model have finite resources, it is assumed that the memory module stores only a limited number of states. To reduce redundancy, only clusters of state–reward pairs are stored. Any clustering strategy may be used, including k-means [52]. Since the decision process is based on similarity, this method maintains its generality and allows for the omission of the problem of “perfect knowledge”, i.e., remembering every detail of an action (since instead of all details, only action representatives are being stored).

This method of storing information also allows for updating the state/reward pairs. If a state is remembered and it appears again with a different reward, the clustering algorithm can recalculate the representative of the action, with a different reward.

#### 3.1.4. Decision Making

The decision module is responsible for choosing between the controllers that will provide the next action: the egoistic or empathetic behavior controller. The first one provides egoistic actions that bring the agent closer to the local goal. The second provides cooperative actions with the same goal but possibly synergistic results. The choice is made based on the reward assigned to the current state of the agent in the egoistic behavior evaluation and, if a signal is received, it considers the reward of empathy target B, calculated by the artificially empathetic behavior evaluation module. The module performs the following comparison:

(5) r(A)≥r(B)

where *A* is the agent’s current state and *B* is the empathy target’s broadcast state. If the r(A) value is higher than r(B), the egoistic behavior of the agent has a greater chance of success in achieving the goal than stopping the current action and helping the neighboring agent. If the contrary is true, acting with empathy may result in a greater chance of success, so the current course of action should be dropped. In the case of multiple incoming signals, only the first one is considered.

#### 3.1.5. Learning

After a full action sequence is performed (i.e., a set of actions resulting in a particular change in environment), the realized action is evaluated. Each agent’s actions are evaluated, based on signals from its neighbors. The sequence is defined as a series of atomic actions that begin with a starting action (the first one without a realized reward) and ends with a last action before an evaluation signal is received:

(6) $$seq_{i}=\left(A_{1}^{i},A_{2}^{i},\ldots ,A_{k}^{i}\right)$$

where $A_{j}^{i}$, j=1,…,k is the state vector and *k* is the number of actions performed before the evaluation signal is received. The realized reward ${\hat{r}}_{i}$ is the first evaluation signal received by the agent, weighted according to the set goal:

(7) $${\hat{r}}_{i}=\hat{A}\cdot W$$

where $\hat{A}$ is the received evaluation signal and the weights *W* are assigned according to the defined agent’s goal. The reward is then assigned to the action process vector, forming new knowledge:

(8) $${\mathrm{ev}}_{{\mathrm{seq}}_{i}}=\left(A_{1}^{i},A_{2}^{i},\ldots ,A_{k}^{i},{\hat{r}}_{i}\right)$$

The aggregated action and the realized reward are then stored in the memory module as newly learned behavior. Given the current action, we find the closest matching action sequence in the knowledge base. Then, if it is similar enough to the current one, we replace the original sequence with its strengthened version. The behavior formulated in this way allows to strengthen the currently existing rules in the knowledge base. If the closest matching action sequence is too far from the current one, a new action sequence is formed.

### 3.2. Simulations

#### 3.2.1. Problem Description

**Figure 2:**
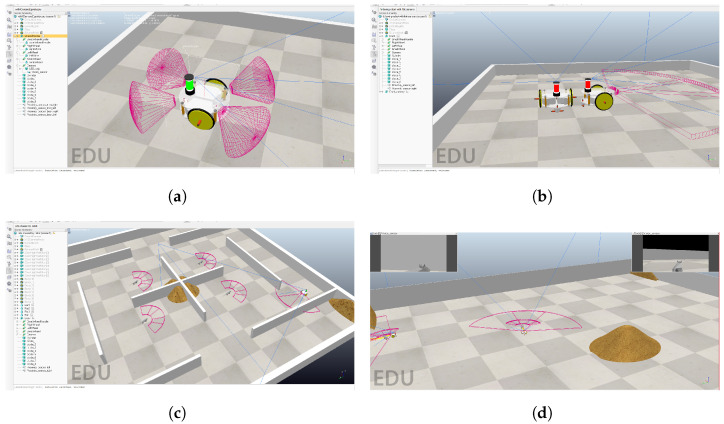
Empathetic swarm simulation in CoppeliaSim. (**a**) Visualization of the agent; (**b**) visual communication; (**c**) warehouse example; (**d**) camera view.

Let us consider the warehouse that stores grain. The stacks of grain often change places due to normal warehouse operations. The warehouse is infested with rats. We introduce a swarm of empathetic guarding robots that collaborate in order to detect rats and find their nests. The robots operate without a central control system but can communicate between themselves. They patrol the dynamic environment. When a rat is spotted, the robot broadcasts a signal containing information about the spotted target, its own chances of success in chasing it, and other information, like the battery level. Robots that receive that signal calculate whether it is better to continue the current action (e.g., patrolling another part of the warehouse, going to the charging station) or to approach the broadcasting robot and assist in chasing the rat. The described environment is an enclosed space with a static obstacle in the form of grain, defined walls, and mobile hostile objects (rats). The robot’s task is to detect and follow the rat, engaging in group encirclement. The rat’s goal is to reach the grain. Different views on virtual experimentation environments are given in Figure 2.

The analysis of robot behavior regarding the influence of artificial empathy was conducted only on chasing robots, as they had the most possible actions to perform and could process the most information among all the robots. In physical experiments, rats were also represented by robots, but they were not equipped with empathetic modules.

Patrolling robots could perform their tasks individually or through communication with other robots. Each robot could signal its own state through an LED strip, displaying information such as the robot class or the currently performed action. This included:

- Call for help;
- Encircling the rat;
- Helping;
- Another robot nearby;
- Rat nearby.

In contrast to the control group, where robots were not equipped with empathetic modules, experiments on empathetic robots indicate that robots have much more information to process before taking specific actions. Similar to the control group, the rat is searched for in the camera image. The empathetic model difference lies in the fact that each patrolling robot additionally signals information about its state and surroundings on the LED strip. It also has the ability to analyze this information from other robots.

This enables robots to make decisions based not only on their own observations but also on those collected from the surrounding environment. Before taking any action, the robot calculates the reward for performing a specific action, i.e., how much it contributes to achieving the global goal. Rewards are calculated for both the currently performed action and the planned action. If the reward for the new action is greater than the currently performed action, the robot interrupts it and starts a new one. Using the artificial empathy module, robots could make decisions that were optimal for the entire group.

The performed experiments considered the following list of scenarios:

- Detection of a rat in the warehouse—solitary pursuit.
  - Robot 1 patrols the warehouse;
  - Robot 1 notices a rat;
  - Robot 1 starts chasing the rat;
  - Robot 1 catches the rat, meaning it approaches the rat to a certain distance.
- Detection of a rat in the warehouse—pursuit handover.
  - Robot 1 patrols the warehouse;
  - Robot 1 notices a rat in the adjacent area;
  - Robot 1 lights up the appropriate color on the LED tower to inform Robot 2 that there is a rat in Robot 2’s area;
  - Robot 2, noticing the appropriate LED color, starts chasing the rat;
  - Robot 2 catches the rat, meaning it approaches the rat to a certain distance.
- Detection of a rat in the warehouse—collaboration.
  - Robot 1 patrols the warehouse;
  - Robot 1 notices a rat;
  - Robot 1 starts chasing the rat;
  - The rat goes beyond Robot 1’s patrol area;
  - Robot 1 lights up the appropriate color on the LED tower to inform Robot 2 that the rat entered its area;
  - Robot 2, noticing the appropriate LED color, continues chasing the rat;
  - Robot 2 catches the rat, meaning it approaches the rat to a certain distance.
- Change of grain color.
  - Robot 1 patrols the warehouse;
  - Robot 1 notices that the grain color is different than it should be;
  - Robot 1 records the event in a report;
  - Robot 1 continues patrolling.
- Change of grain color—uncertainty.
  - Robot 1 patrols the warehouse;
  - Robot 1 notices that the grain color is possibly different than it should be—uncertain information;
  - Robot 1 lights up the appropriate color on the LED tower;
  - Robot 2, noticing the appropriate LED color, expresses a willingness to help and approaches Robot 1;
  - Robot 2 from the adjacent area checks the grain color and confirms or denies Robot 1’s decision;
  - Robot 1 records the event in a report if confirmed by Robot 2;
  - Robot 2 from the adjacent area returns and continues patrolling;
  - Robot 1 also continues patrolling.
- Weak battery.
  - Robot 1 has a weak battery;
  - Robot 1 lights up the appropriate color on the LED tower, expressing a desire to recharge its battery;
  - Robot 2, noticing the appropriate LED color, agrees to let Robot 1 recharge the battery;
  - Robot 1 goes to recharge;
  - Robot 2 additionally takes over Robot 1’s area for patrolling.
- Exchange of patrol zones.
  - Robot 1 passes through its patrol area several times without any events;
  - Robot 1 lights up the appropriate color on the LED tower, expressing a desire to exchange the patrol area;
  - Robot 2, noticing the appropriate LED color, expresses a desire to exchange the patrol area;
  - Robot 1 and Robot 2 exchange patrol areas.

#### 3.2.2. Implementation

The simulations were performed in CoppeliaSim (V4.4.0). Up to ten robots, equipped with virtual cameras, LED communication, and touch sensors were to detect and chase four rats in a synthetic warehouse environment. Robots broadcast state signals to other agents, which receive them via camera and decide whether to take egoistic or empathetic action.

The YOLOv2 real-time object detection system [53] was used to detect objects in the camera images, and the VGG16 [54] convolutional neural network was used to determine the status of other robots (sent via LED strip). All simulation scripts were implemented in Lua (internal CoppeliaSim scripting) and Python (external backend service).

All the fuzzy descriptions like “far” or “long” are modeled with linguistic variables and terms—the value of a parameter is actually the value of a membership function for each of the considered terms.

Vectors of parameters in Table 1 and Table 2 are used as initial knowledge. Each new state that arises is compared to those, and the similarity is calculated to decide if the new state has a chance of success or not. Here, we use the similarity measure:

(9) $$s\left(A^{j},A^{i}\right)=1-\frac{\sqrt{\sum_{k=1}^{n}{\left|x_{k}^{j}-x_{k}^{i}\right|}^{2}}}{n}\;.$$

#### 3.2.3. Simulation Results

Robots cooperate in order to detect and chase the rats, and empathetic behaviors are visible. Due to the limitations of CoppeliaSim, mainly the lack of repeatability and poor performance when using virtual cameras, simulations did not allow for a reliable comparison between egoistic and empathetic behaviors. These problems lead directly to the OPEP project. OPEP allows for the inclusion of such factors as acceleration, friction, light intensity, and reflections, while maintaining high control over the environment and experiment course.

During the simulation, the time in which the robots achieve the global goal, i.e., detecting and catching all rats, was measured. For each case, empathic and non-empathic models, 10 experiments were conducted, measuring the time to achieve the global goal. An important aspect was that objects in the arena were randomly distributed each time to ensure diversity in the observed behaviors.

After conducting experiments on a swarm of five robots, it was decided to double the number of objects in the scene. This change introduced more opportunities for interactions between individual units, and the simulation could proceed differently. Additionally, the larger the group of patrolling robots, the more the positive impact of empathic behaviors can be observed, allowing for a focus on the analysis of behaviors between neighboring objects.

As in previous experiments, objects before each simulation were randomly distributed, and the simulation ended when the global goal was achieved, i.e., when all rats were detected and surrounded.

In this way, a total of 40 experiments were conducted in 4 variants. This material was further analyzed, with a primary focus on the analysis of model behaviors using artificial empathy and those without it. In many cases, the empathic model recorded lower times to achieve the global goal. However, the differences are small, with the effectiveness of empathy being the most visible in larger groups. In such situations, the true power of unit cooperation, forming the entire swarm, can be observed.

An interesting phenomenon was the significant differences in times between individual simulations. The shortest simulation time for five patrolling robots was only 58 s, while the longest was as much as 116 s, nearly a twofold difference. The average simulation time in the egocentric model was 84.1 s, and in the empathy-utilizing model, it was 81.6 s. Summing up all experiments in this section, the empathetic swarm of robots performed its tasks, on average, 2.5 s faster. For 10 patrolling robots, the fastest achievement of the goal occurred after 88 s, while the longest took 205 s. In this case, there were many more possible interaction scenarios for 10 robots, influencing the disparities in simulation times. The average neutralization time for all viruses in the egocentric model was 148.5 s, while for the empathetic model, it was 137.3 s. The significant positive impact of using the artificial empathy module is evident, with a difference of 11.2 s, confirming the effectiveness of the empathetic model. Unfortunately, a more accurate and reliable statistical analysis is not possible. This is due to the huge discrepancies between repetitions and the influence of many uncontrolled random factors resulting from the CoppeliaSim simulator on the experiment.

We prepared a few visualizations of the proposed empathetic model, along with a comparison with the egoistic one (videos available on https://github.com/open-pep/coppelia-simulations accessed on 13 December 2023).

1. Egoistic, two rats. Shortly after starting a patrol, both robots spot the same rat and start chasing it. Meanwhile, the second rat destroys the grain located in the middle of the arena. After neutralizing the first rat, one of the robots begins chasing the second pest.
2. Empathetic, two rats. The robot on the right spots a rat and signals it with an LED strip. The second robot, noticing this, continues to patrol the surroundings in search of other pests. After a while, it detects the second rat and starts following it. As a result, both rats are neutralized and grain loss is reduced.
3. Egoistic, robots run out of battery. Robots detect the same rat. During the chase, the robots interfere with each other, making it difficult to follow and neutralize the rat. Eventually, the rat is neutralized, but before the robots can spot and begin their pursuit of the other pest, both of them run out of battery and the second rat escapes.
4. Empathetic, low battery help. The robot on the right starts chasing the detected rat. During this action, the agent signals with an LED strip that it needs assistance, due to a low battery level. The other robot notices this and decides to help to catch the weaker rat. After neutralizing it, the second robot starts searching for other pests.

**Figure 3:**
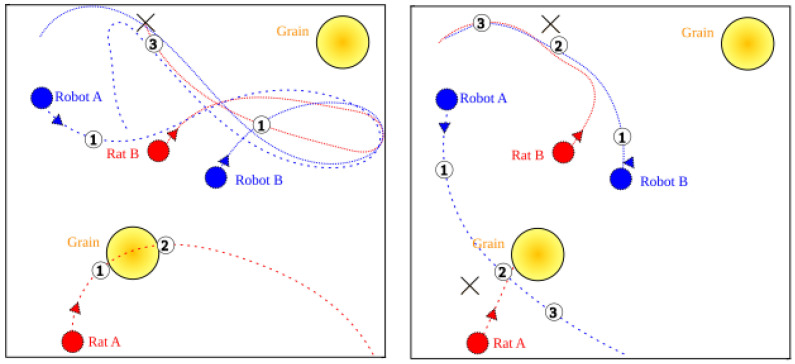
The movement trajectories of all agents for egoistic (**left**) and empathetic (**right**) visualisations. The numbered points in the figure depict points in time. (**Left**) (1) Both robots spot Rat B while Rat A starts to damage grain. (2) Rat A starts searching for a new target. (3) Both robots finally neutralize Rat B. (**Right**) (1) Both robots spot rats, due to cooperation, they start to chase different targets. (2) Both robots start neutralizing rats. (3) After neutralizing the rats, both robots start to patrol the area.

Two initial visualizations are summarized in Figure 3. The movement trajectories of all agents are shown by a dashed and dotted lines. It can be seen that in the egoistic variant, both robots undertake the pursuit of the same rat. While in the empathetic variant, thanks to communication, the robots started a chase after both rats, so that the grain resources were not damaged.

### 3.3. Open-Source Physical-Based Experimentation Platform

In the field of robotics, experiments are crucial for the development and verification of new algorithms and technologies. However, conducting experiments in this area is challenging, costly, and comes with a range of problems.

Simulations—one of the ways to conduct experiments—are often simplified and inaccurate due to the multitude of parameters that need to be considered. On the other hand, accurate simulations are very time-consuming. It is difficult to include all parameters in the simulation, as some may be unidentified or challenging to model. CoppeliaSim is one tool used for simulating the motion of robot swarms, but it has significant limitations and does not consider all parameters of the real environment, leading to unrealistic simulation results. Time-consuming robot swarm simulations also pose a problem, making it difficult to test various scenarios and restricting frequent algorithm changes.

Real-world experiments, on the other hand, are costly, requiring the purchase of components and the creation of physical experimental platforms, which is time-consuming. However, experiments using dedicated hardware lack high repeatability and reproducibility of results, making it challenging to compare models experimentally. Additionally, there is a lack of standards and a unified approach to the design and implementation of mobile robots, requiring individualized approaches for each experiment and resulting in significant time delays and increased costs. These issues exacerbate discrepancies between simulations and experiments.

Therefore, in this work, we propose the concept of the open-source physical-based experimentation platform (OPEP), which is an intermediate environment between simulators and full experiments on dedicated equipment. In the following, we will present general assumptions toward the offered functionalities, the architecture, and the implementation of two versions of prototypes of the postulated solution.

#### 3.3.1. Proposed Platform Features and Architecture

**Figure 4:**
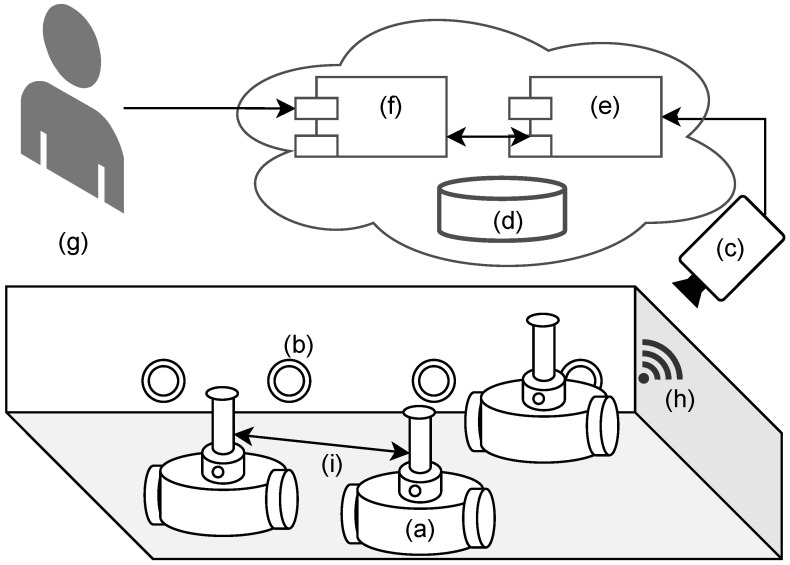
Context architectural diagram for OPEP. (a) Independent robots that form a swarm; (b) automatic charging stations; (c) overhead controller (camera + miniPC); (d) data store; (e) experiment control API module; (f) simulation web API; (g) researcher interacts with the system via a web browser; (h) wireless communication with overhead (maintenance only); (i) visual communication between robots.

The open-source physical-based experimentation platform (OPEP) consists of several parts—a swarm of autonomous, mobile robots, an arena for controlled experiments, charging stations, an overhead controller (camera and mini PC), and a web application for remote experiment control, as depicted in Figure 4. The platform allows for performing experiments on physical robots remotely, in a controlled environment. It stands as a middle step between fallible and imperfect simulations and costly physical implementation.

The proposed experimentation platform contributes to the creation of a completely new product: a universal, remote service for experimenting with and testing artificial intelligence algorithms on a hardware swarm of robots, provided in a cloud computing model. Its main features are outlined in the following paragraphs.

1. Comprehensive support for swarm design process using hardware platform. This feature corresponds to the need to verify AI algorithms in a hardware environment, including early-stage development, consideration of environmental parameters that are unavailable in simulations, and the ability to study algorithms, considering variable environments and interactions. In this area, there are two alternatives: comprehensive algorithm evaluation (Kilogrid + Kilobots, DeepRacer) and simulation software (CoppeliaSim v4+, DynaVizXMR, EyeSim v1.5+, Microsoft Robotics). Alternative solutions only support the design process in simulated environments or require significant financial investments for prototyping, limiting accessibility in early development stages.
2. Low cost of building and size of swarm robots. This feature corresponds to the need to evaluate complex behaviors and the latest AI algorithms in a large swarm of robots, considering the requirements for low cost and easy availability of solutions. Alternatives include miniature robots like Kilobots and mini sumo robots. Those solutions are expensive, with costs often including additional resources and services. Additionally, computational power drastically decreases with the robot’s size, limiting capabilities such as running a vision system.
3. Remote programming of robots. This feature addresses the need to share research/educational infrastructure without physical access, fostering interdisciplinary and international research collaborations. Alternatives include cloud-based robot simulators like AWS Robomaker and DeepRacer. In competitive solutions, this functionality is only available in simulations or limited environments and specific research areas.
4. Standardization and Scalability of experimental environment. This feature corresponds to the need to adapt and expand the experimental platform to different projects while maintaining standardization for experiment repeatability and reproducibility, facilitating comparisons across research centers. Alternatives include open-source software and hardware projects like SwarmUS, Kilobots, colias.robot, as well as simulation software. They lack the ability to expand robot software in any way using high-level languages. Moreover, existing solutions are not designed for result repeatability (e.g., randomness in Kilobots’ movements).
5. Open specification and hardware. This feature corresponds to the need for independently building a complete experimental platform. Current solutions include open-source software and hardware projects, such as SwarmUS, Kilobots, and colias.robot. Most competitive solutions are closed, and open solutions often have limited computational resources.

#### 3.3.2. Platform Implementation

##### First Prototype

**Figure 5:**
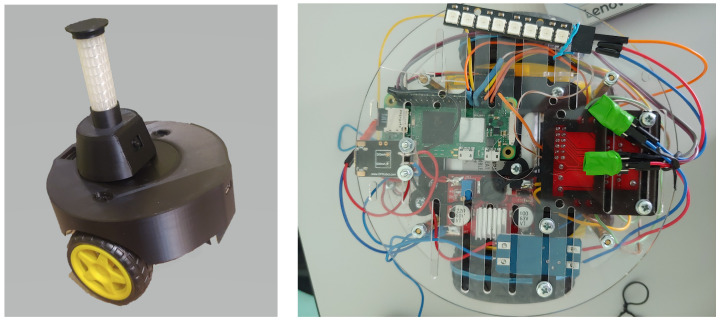
Hardware implementation and view on internal components of the first prototype.

**Figure 6:**
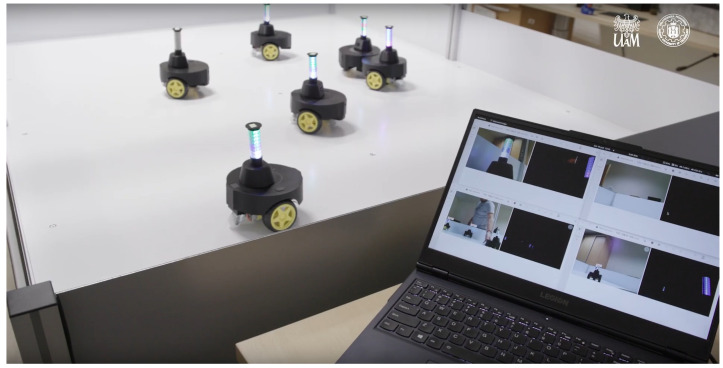
First version prototypes moving on the arena with the live monitoring of cameras.

The authors conducted a series of analyses and experiments, creating a swarm of 8 early prototypes of robots equipped with mobility, vision, and communication systems, enabling the realization of simple empathetic behaviors. The prototype robots had a diameter of 14.9 cm and a height of 15 cm, and were equipped with a 4000 mAh Li-Poly battery, allowing about 40 min of continuous operation (Figure 5 and Figure 6). Based on team member experiences during the creation of the prototype robot swarm, several phenomena and issues crucial for proper robot operation in a real environment were observed. These would likely be overlooked in computer simulations, including mirror reflections of robots in the arena walls, slipping robot wheels, uneven traction, imperfections in mechanical components (e.g., motors, gears), and the impact of the environment on the performance of the vision system (too strong or weak room lighting).

The first versions of robots were built with the use of Raspberry Pi Zero 2 W microcomputers that took care of robot control, vision, and all decision-making. Agents used two motors to power the wheels. The communication was performed via a custom 360° RGB LED strip, placed on a rotating turret, which also held the OV5647 5MPx camera, which gave an effective 240° angle of view. The use of 8 RGB LED lights allowed expressing about 2 M distinguishable inner states. The communication was imperfect since robots had to detect the signal that could be disturbed by light level, reflection, other robots in view, etc. The outer case was 3D-printed and was designed for minimal collision damage.

Each robot in the swarm runs on 64-bit Raspberry Pi OS Lite (5.15 kernel). The vision system is implemented using *picamera2* (0.1.1) and *opencv-python* (4.6.0.66) libraries. Intra-process communication is handled using Redis (7.0.5) pub/sub feature. Web-based user interface is still being actively developed. It uses Python 3 and Go 1.19 for API implementation. The overhead controller uses both visual monitoring (experiment recording) as well as a wireless network (software upload, swarm maintenance). The ongoing research and development of the project can be tracked on GitHub [55] (the project is in the process of migrating from an internal repository to GitHub and not all components are available yet).

##### Second Prototype

The first prototype has some problems that need to be solved so that more effective research can be conducted:

- The robots need to be stopped and physically plugged in for charging when the batteries run out. This causes delays in conducting experiments.
- Current robots are characterized by large dimensions compared to the work area. This minimizes the simultaneous number of robots that can move around the arena.
- The presence of an experimenter is required to activate the robots. This makes it impossible to conduct remote experiments.

For the development of the next, more efficient, and miniaturized iteration of the platform, it is necessary to solve the above problems by creating a custom, compact version of the PCB. This requires conducting a thorough analysis of available circuit boards and selecting the best solutions to use in designing the electrical as well as mechanical structure of the robots that make up the swarm. This will allow the robots to respond to their environment in the best possible way, and allow researchers to conduct experiments on artificial empathy remotely.

**Figure 7:**
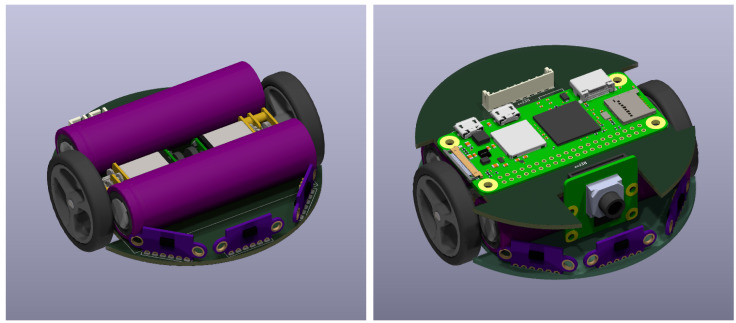
Visualization of the second prototype, with (**left**) and without (**right**) the upper board.

Due to the minimization of the robot’s dimensions, its design envisions two circuit boards connected above each other. The lower board will be responsible for interacting with the environment, and the upper board will be responsible for information processing and decision-making. The second version of the prototype is less than 8 cm in diameter and has a much smaller height (Figure 7).

**Figure 8:**
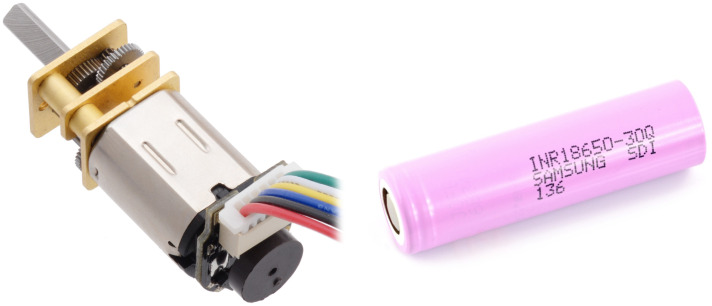
Engine and battery used in the second prototype.

The robot’s movement will be accomplished using two miniature-geared motors manufactured by Pololu (Figure 8). Each is equipped with a magnetic encoder based on TLE4946-2K Hall effect sensors, specifically designed for this scenario. The rotation of the motors will additionally be monitored by current sensors, one for each motor, to achieve accurate speed control. To eliminate losses on typical shunt resistor current sensors, a circuit that measures the current based on the Hall effect can be used. Examples of such sensors are the ACS712 or ACS724. To power the motors, DRV8833 will be used as an executive circuit. This controller enables the operation of two motors and is characterized by its requirement of only two control lines per motor to regulate the speed and direction of rotation. Additionally, it is easy to operate using hardware Timers in the microcontroller.

To provide the robot with precise orientation in the field, it will be equipped with an integrated BNO055 chip, consisting of a Bosch accelerometer, gyroscope, and magnetometer. This chip will support the robot’s motion algorithms for maximum precision in its maneuvers. In addition, the VL53L0X sensors will provide information about the distance of obstacles in front of the robot. The use of these sensors introduces increased complexity in both the board design and control algorithms compared to traditional push buttons and limiters. Nevertheless, this change contributes to reducing mechanical contact with the environment, which translates into minimizing the risk of damage to the robot and increasing its reliability.

Two Samsung INR18650-35E lithium-ion cells with a total capacity of 7 Ah will be used to power the robot (Figure 8). Due to the robot’s small size, the design will utilize cylindrical cells instead of flat lithium-polymer batteries, necessitating the addition of protection circuits (Figure 7). In order to ensure safe power management, a DW01 battery protection circuit will be employed in conjunction with the executive transistors. This circuit is designed to monitor the battery voltage to prevent exceeding the permissible range and to disconnect the voltage in case of a short circuit. The charging process will be supervised by the TP5100 chip, which not only provides up to two amps of current but also ensures that the cell charging process adheres to the constant current constant voltage (CC CV) charging specification. One of the project’s goals is to enable the robot to charge without human intervention. To achieve this, two contact pads will be placed on the bottom of the lower circuit board. Through these, the robot will be able to charge its batteries by hovering over a special charging station. This solution is extremely simple, occupies minimal space, and does not introduce energy transfer losses.

The robot, despite its small size, will be equipped with systems that require high power consumption. To meet their expectations, the project will use two inverters, one with an output voltage of 5 V—U3V70A, which is capable of supplying up to 10 A at peak demand even for a few seconds, and the second inverter, this time for circuits powered by 3.3 V—U7V8F3, will provide the currents for all sensors and the microcontroller. It is worth noting that the 3.3 V inverter will operate in two modes: step-down when the battery is charged and step-up when the battery is closer to being discharged.

The bottom board will also house a 32-bit microcontroller from the STM32 family. Its high computing power, flexibility, and popularity will allow the implementation of almost arbitrarily complex algorithms. It will control all the above circuits and communicate with the robot’s main processor.

**Figure 9:**
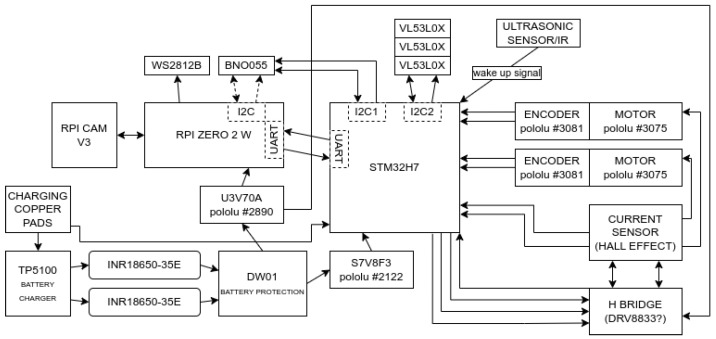
Block diagram showing the internal layout of the second prototype.

The role of the device’s brain will be played by the Raspberry Pi Zero 2W; it is a quad-core, single-board computer on a top circuit board, which will react to its environment and other robots with the help of the Raspberry Pi Cam V3. All artificial intelligence and empathy algorithms will be implemented right on this microcomputer. Signaling of its own internal state will be achieved by individually addressable RGB LEDs—WS2812B, arranged in 8 rows, each with 3 LEDs. This will make it possible to display at least one and a half million different states. A detailed diagram showing the internal layout of the proposed device is shown in Figure 9.

##### Experimentation Arena

**Figure 10:**
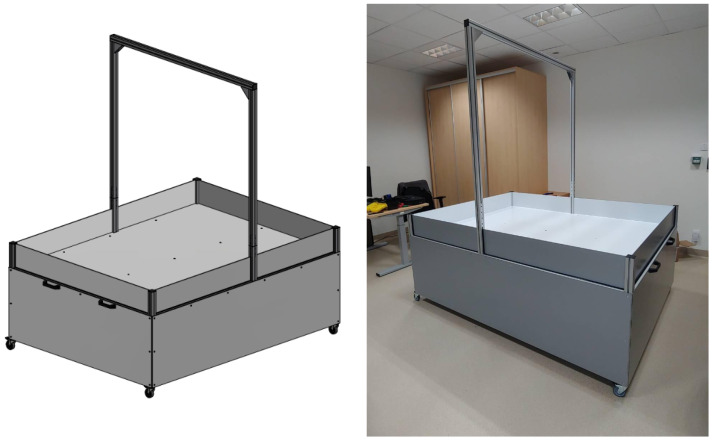
Visualization and realization of the controlled environment for the empathetic robot swarm arena.

One of the challenges was to create a suitable arena that serves as an environment for controlled experiments (Figure 10). Another still not fully solved challenge is the inability to modify and introduce variability to experimental platforms, limiting the number of test scenarios and hindering research in different applications. This arena was designed to simulate various environmental conditions and allow the study of swarm behavior in different situations, added to the arena as modular modifications (e.g., obstacles).

This will allow testing whether and how (with what energy expenditure and difficulties) the robot can perform simple tasks in the presence of designed variable elements in the environment. Conducting these experiments will provide insights into the requirements for the experimental arena and robot prototypes, allowing for further considerations in subsequent prototyping iterations.

## 4. Conclusions

This paper proposes a model for swarm behavior control through artificial empathy, using the fuzzy set theory and similarity measures. The model emulates human empathetic decision processes to optimize behavior toward achieving a common goal. The authors’ main contribution is redefining the theoretical cognitive empathy model into a particular swarm control model, with a significant focus on knowledge representation and decision-making processes.

Our model differs from existing swarm models of artificial empathy, such as [36,41], in that it incorporates empathetic communication, knowledge representation, and the use of similarity measures to convey empathy mechanisms. Unlike those models, which utilize a simple parameter to describe the empathy target’s state, our approach is more comprehensive and effective. This approach is inspired by the emotional/cognitive empathy model in neural science and filters agent-broadcasted states based on similarity to known situations.

The example presented here demonstrates the model’s versatility across platforms, emphasizing only a fraction of its applicability. The model’s independence from a specific application allows for the separate development of empathetic decision-making modules and platform controllers. It is worth noting that the model could be applied in dynamically changing environments, where agents can ’borrow’ knowledge from others. Incorporating the perceived rewards of other agents into the decision-making process could enable neighboring agents to learn from experience by comparing differences in perceived and realized rewards. This paper lays the groundwork for further exploration and development of empathetic swarm control models in diverse environments.

The integrated experimental environment proposed in the article is an important step in the development of research on swarm robot control algorithms. This, in turn, will increase the possibility of implementing the results of scientific research in practice. The proposed experimental platform, its scope, and architecture are based on the experience that has been developed while working on two of its earlier prototypes. As a result, it was possible to optimize many technical and operational parameters of the developed solution. It is also not insignificant that the work on the hardware environment is strongly connected with research on empathetic robotic swarms, imposing real requirements on the direction of the project’s development. The most significant example of this is the use of innovative vision communication using LED towers.

In further research, we want to upgrade the model to more accurately recreate human empathy. The egoistic behavior evaluation module will be implemented using a neural network, and the similarity of other agents’ states will be determined based on internal knowledge represented by the net. Also, reinforcement learning is to be used to teach the net new data and adapt to the environments and behaviors of other agents.

Our future work will aim to validate the artificial empathy model as an effective learning method by simulating empathetic behavior in isolation from the physical layer of the robots in the swarm. Further research should express the proposed learning model in the language of reinforcement learning and use the tools available in this area to obtain reproducible quantitative results that demonstrate the effectiveness of empathetic methods.

The variability of the experimental environment is a distinctive feature of the proposed experimental platform. Research efforts will be necessary to identify available and feasible strategies to achieve this goal. Further research will consider elements of a variable environment, such as changing parameters (e.g., lighting) and altering the structure of the arena (obstacles, cooperative logical puzzles). A key assumption is the automation of the process for introducing modifications to the arena. This is crucial for achieving repeatability in experiment results and enabling remote access to the platform.

## Figures and Tables

**Table 1:** Parameters describing the state of the agent.

Name	Sym	Description of Boundary Values
others close	a	1 many other agents in the vicinity, 0 for none
in touch	n	1 for long contact time, 0 for none
long search	t	1 for the long duration of the current search, 0 for not searching
calling for help	c	1 for calling for a long time, 0 for not calling
neutralized	e	1 if “I am inactive” signal was received from the newly inactive agent; 0 if not
close to neighbor	d	1 if the distance to the neighbor is 0; 0 if the distance to the neighbor is far
target at right	p	1 for the agent at the immediate right, 0 for the agent not in sight
target at left	l	1 for the agent at the immediate left, 0 for the agent not in sight
fully charged	f	1 for the fully charged robot, 0 for not charged
helping	h	1 for the long duration of helping, 0 for not helping
reward	$s_{i}$	describes the chance of success of the current action sequence

**Table 2:** Agent’s initial knowledge—states and rewards.

Parameter	$x_{1}$	$x_{2}$	$x_{3}$	$x_{4}$	$x_{5}$	$x_{6}$
a	0.5	1.0	0.5	0.5	0.0	0.1
n	0.5	0.5	0.5	0.0	1.0	1.0
t	0.5	0.5	0.5	0.5	0.5	0.5
c	1.0	1.0	1.0	0.5	1.0	0.5
d	0.5	1.0	0.5	0.5	0.0	0.1
p	0.5	0.5	0.5	0.5	0.5	0.5
l	0.5	0.5	0.5	0.5	0.5	0.5
f	0.5	0.5	0.5	0.5	0.5	0.5
h	0.5	0.5	0.5	1.0	0.5	1.0
$r_{i}$	1.0	1.0	1.0	1.0	0.0	0.0

## Data Availability

Data are contained within the article.
